# Deep-learning-based information mining from ocean remote-sensing imagery

**DOI:** 10.1093/nsr/nwaa047

**Published:** 2020-03-19

**Authors:** Xiaofeng Li, Bin Liu, Gang Zheng, Yibin Ren, Shuangshang Zhang, Yingjie Liu, Le Gao, Yuhai Liu, Bin Zhang, Fan Wang

**Affiliations:** Key Laboratory of Ocean Circulation and Waves, Institute of Oceanology, Chinese Academy of Sciences, Qingdao 266071, China; Center for Ocean Mega-Science, Chinese Academy of Sciences, Qingdao 266071, China; College of Marine Sciences, Shanghai Ocean University, Shanghai 201306, China; State Key Laboratory of Satellite Ocean Environment Dynamics, Second Institute of Oceanography, Ministry of Natural Resources, Hangzhou 310012, China; Key Laboratory of Ocean Circulation and Waves, Institute of Oceanology, Chinese Academy of Sciences, Qingdao 266071, China; Center for Ocean Mega-Science, Chinese Academy of Sciences, Qingdao 266071, China; College of Oceanography, Hohai University, Nanjing 210098, China; Key Laboratory of Ocean Circulation and Waves, Institute of Oceanology, Chinese Academy of Sciences, Qingdao 266071, China; Center for Ocean Mega-Science, Chinese Academy of Sciences, Qingdao 266071, China; Key Laboratory of Ocean Circulation and Waves, Institute of Oceanology, Chinese Academy of Sciences, Qingdao 266071, China; Center for Ocean Mega-Science, Chinese Academy of Sciences, Qingdao 266071, China; Key Laboratory of Ocean Circulation and Waves, Institute of Oceanology, Chinese Academy of Sciences, Qingdao 266071, China; Dawning International Information Industry Co., Ltd., Qingdao 266101, China; Key Laboratory of Ocean Circulation and Waves, Institute of Oceanology, Chinese Academy of Sciences, Qingdao 266071, China; Center for Ocean Mega-Science, Chinese Academy of Sciences, Qingdao 266071, China; Key Laboratory of Ocean Circulation and Waves, Institute of Oceanology, Chinese Academy of Sciences, Qingdao 266071, China; Center for Ocean Mega-Science, Chinese Academy of Sciences, Qingdao 266071, China

**Keywords:** ocean remote sensing, big data, artificial intelligence, image classification

## Abstract

With the continuous development of space and sensor technologies during the last 40 years, ocean remote sensing has entered into the big-data era with typical five-V (volume, variety, value, velocity and veracity) characteristics. Ocean remote-sensing data archives reach several tens of petabytes and massive satellite data are acquired worldwide daily. To precisely, efficiently and intelligently mine the useful information submerged in such ocean remote-sensing data sets is a big challenge. Deep learning—a powerful technology recently emerging in the machine-learning field—has demonstrated its more significant superiority over traditional physical- or statistical-based algorithms for image-information extraction in many industrial-field applications and starts to draw interest in ocean remote-sensing applications. In this review paper, we first systematically reviewed two deep-learning frameworks that carry out ocean remote-sensing-image classifications and then presented eight typical applications in ocean internal-wave/eddy/oil-spill/coastal-inundation/sea-ice/green-algae/ship/coral-reef mapping from different types of ocean remote-sensing imagery to show how effective these deep-learning frameworks are. Researchers can also readily modify these existing frameworks for information mining of other kinds of remote-sensing imagery.

## INTRODUCTION

The ocean accounts for about 71% of Earth's surface. Humans had minimal ocean observations before the Seasat, the first Earth-orbiting satellite designed for remote sensing of Earth's oceans, launched in 1978 [1]. Although Seasat only operated for 105 days, the sensors on board Seasat had acquired much more data about the vast ocean than all previous sensors combined. Such high-efficiency data collection stimulated the fast development of ocean-satellite remote sensing. Since then, more and more satellites carrying microwave, visible, infrared sensors have been launched to measure various ocean physical, biological and other parameters that lead to significant improvement in our understanding of the ocean during the last 40 years [2–7].

**Figure 1.**
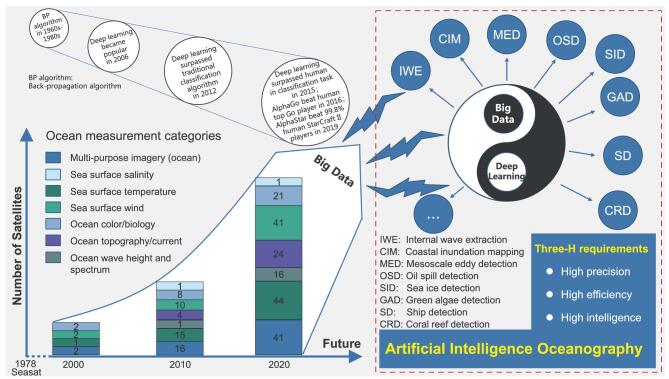
Ocean remote sensing has entered into the big-data era with the rapid increase in on-orbit satellites, sensors and data-archive volume. Ocean remote-sensing big data can offer abundant data fuel to data-driven deep learning, while data-driven deep learning provides a promising way to make the best of ocean remote-sensing big data. The win–win combination of them will make future ocean remote sensing more precise, efficient and intelligent. The numbers of sensors for the different ocean-measurement categories were calculated from the data of the Committee on Earth Observation Satellites (CEOS) database (http://database.eohandbook.com/measurements/categories.aspx?measurementCategoryID = 3).

There are two types of remote-sensing sensors: active and passive sensors. The active sensors measure sea-surface height or SSH (altimeter), sea-surface roughness (synthetic-aperture radar or SAR), sea-surface wind (scatterometer, SAR). In contrast, the passive sensors measure sea-surface salinity, sea-surface temperature (SST) and water-leaving radiance using microwave/infrared radiometers and optical sensors. According to the report from the Committee on Earth Observation Satellites (CEOS), for each primary ocean-surface parameter, there are currently a dozen satellites on-orbit making the daily measurements (Fig. 1). Other tens of satellites have also been approved or planned over the next 20 years. The increase in satellite numbers has resulted in a rapid rise in the volume of ocean-satellite data archives that number tens of petabytes. Also, due to the improvement of spatial, temporal and spectral resolutions of various sensors, the variety of ocean-satellite data now increases. Ocean remote sensing now has the typical five-V (volume, variety, value, velocity and veracity) characteristics of big data.

A dilemma is that big data do not always guarantee that people can get more valuable information from them because the useful information is usually sparsely hidden in massive ocean-satellite data. In the past 40 years, many efforts have been put into the tasks to develop and validate retrieval algorithms to generate a long-time series of many standard global ocean parameters [6–8]. Currently, we need efficient and even intelligent approaches to improve information-extraction capability and efficiency with emergent powerful deep-learning (DL) technology. We need to strengthen our skills in three aspects. First, some ocean phenomena like internal waves and algal blooms are locally generated and their signatures only consist of a tiny percentage of an ocean remote-sensing image. We cannot extract this type of information as we do for the standardized ocean-parameter (SST, etc.) products from the direct measurements of satellites. Second, there is much essential information hidden in these long-time series data that requires new data-driven information-mining algorithms. Besides, extracting such information from a high-rate downlink satellite data stream requires high-speed data processing. Deep-learning-based approaches can satisfy all these requirements.

Traditionally, we can categorize information extraction in ocean remote-sensing images into two types: supervised classification and object detection. Supervised classification means classifying images or pixels, usually referring to samples, according to given classes. Pixel-level supervised classification, also named supervised semantic segmentation, is more often encountered in ocean-satellite-image applications. Oil spills, sea ice, eddy and algal blooms usually have discriminable patterns in satellite images with irregular shapes [9]. Extraction of such information is an essentially supervised semantic segmentation that includes light-spectrum combination, polarization decomposition, co-occurrence matrices, spectrum analysis (e.g. wavelets) and other methods. Object detection in ocean remote-sensing imagery usually refers to detecting objects (ships, oil rigs, etc.) that are distinguished from the surrounding image backgrounds. A constant false-alarm rate (CFAR) is the most common statistical approach for ship detection in ocean remote-sensing images [10]. The methods work but may not be optimal for a specific end-to-end (data-to-information) problem, since the traditional supervised classification and object-detection approaches do not consider spatial structure features or use the features extracted by human-designed operators.

An artificial intelligence (AI) method can help to get better and fast results. AI based on an artificial neural network (ANN) computational model that can learn the relationship among its inputs and outputs from given training samples. Biological neural networks initially inspired ANN development in the 1940s. Since then, ANN has advanced from the simple McCulloch-Pitts and Perceptron models of the 1950s–1960s and the back-propagation algorithm was developed in the 1960s–1980s (Fig. 1). The introduction of convolutional layers and pooling layers occurred in the 1980s. And the popular deep neural networks (DNNs) did not start until the early 2000s (Fig. 1). The introduction of convolutional layers primarily reduces the number of network parameters by local-linking and weight-sharing, while pooling layers reduce the size of the feature maps by down-sampling [11–13]. Please note that a DNN is different from a convolutional neural network (CNN) in concept. DNN means the neural network architecture is deep and complex, while CNN means convolutional layers are used in the neural network. When a DNN contains convolutional layers, it is also a CNN. When a CNN is deep, it is also a DNN. A deep structure with alternation of convolutional and pooling layers gives DNNs the powerful capability to efficiently extract abstract features from images. DNN training is to find the optimal structure and coefficients based on a large number of labeled samples. Once trained, a DNN can better extract the data features than the traditional approaches that use human-designed rules and then infer the information behind the data. DNNs achieved significant superiority over traditional classification approaches in the ImageNet Large-Scale Visual Recognition Challenge (ILSVRC) 2012 and dominated the ILSVRCs in the following years. In 2015, DNNs achieved better performance (4.94% top-5 error) than human (5.1% top-5 error) on the 1000-class ImageNet 2012 classification data set [14] (Fig. 1).

U-Net is a representative DNN for semantic segmentation [15,16]. U-Net has the encoder–decoder structure and the skip connection between the encoder and the decoder, and these make U-Net have excellent performance. The DNNs for object detection can be divided into one-stage networks (e.g. single-shot multi-box detector (SSD)) and two-stage networks with a new network branch for initial screening (e.g. Faster-RCNN) [17,18]. Generally, one-stage networks have higher computational efficiency because of simpler designs. Essentially, there is no difference in information extraction between ocean remote sensing and conventional images. Therefore, powerful DNNs also have massive potential for mining information from ocean remote-sensing data and, conversely, ocean-satellite big data can provide data to fuel the DNNs.

As shown below, the information extraction of ocean remote-sensing data is undergoing the evolution from human-designed rule-based models to end-to-end learning models in the big-data era. Most of the previous ANN models applied in ocean remote sensing were based on fully connected neural networks (FNNs). The critical shortcoming of FNNs is their inefficiency in processing high-dimension data, including extracting contextual features from images. Therefore, a remedy was often made in previous FNN-based classifications by increasing an additional preprocessing step to obtain contextual features as inputs of FNN using human-designed rules. For instance, people need to extract textural features of ocean remote-sensing images in oil spills and sea-ice classification. This additional step is called feature engineering in the field of machine learning. Since this extra level uses human-designed rules, the FNN-based classification models are still not end-to-end models. The powerful capability of the feature extraction of DNNs can overcome this problem. Recent studies have shown that DNNs have achieved excellent performance in information extraction from the ocean remote-sensing data of oceans and others, although both opportunities and challenges still exist [19].

AI oceanography development is just in its infancy. And potential deep-learning applications in the oceanography field urgently need to be widely studied. Besides the classification and semantic-segmentation tasks mentioned above, deep-learning models as well as other AI models can also find their positions in observational data fusion, parameter retrieval, forecast, etc. of the ocean and atmosphere [7,20–24]. In very recent times, a deep-learning model has successfully made skillful forecasts of El Niño/Southern Oscillation, showing great potential in solving this complex scientific problem [24]. Other new advances were also achieved in applying deep-learning models to make short-term forecasts of tropical cyclone tracks and intensity [22,23].

The deep-learning frameworks can be blended. The blended models can achieve more complex functions and have improved accuracy and increased efficiency. We can also combine the deep-learning model with a physical equation-based numerical weather-prediction model. The potential combinable aspects include quality control, parameterization, post-processing, etc. Application of DL technology in weather and ocean forecasting is an up-and-coming research area, as it is possible to fulfill the advantages of both DL and numerical modeling jointly. Recent work in the research area has been well reviewed by Boukabara *et al.* [23]. Additionally, ensemble learning for improving classification performance in the field of machine learning can also be used to combine DL models and scientists have been exploring some in remote-sensing retrievals and ocean forecasts [7,25–27].

In this review paper, we first systematically review two deep-learning frameworks that carry out ocean remote-sensing image classification and then present eight typical applications in ocean internal-wave/eddy/oil-spill/coastal-inundation/sea-ice/green-algae/ship/coral-reef mapping from different types of ocean remote-sensing imagery to show how effective these deep-learning frameworks are (Fig. 1).

## DNN FRAMEWORKS OF CLASSIFICATION AND DETECTION

As mentioned above, in order to extract sparse but valuable information from a large volume of ocean remote-sensing data, we need to construct end-to-end DNN models. There are two basic tasks to mine multidimensional, or even multi-source, ocean remote-sensing data—that is, pixel-level classification and object-level detection. The applications presented in the next section can be categorized into these two tasks, although they may have different conventional terms, such as internal-wave-signature extraction, coastal-inundation mapping and mesoscale-eddy detection. In this review, for the pixel-level classification and object-level detection tasks in ocean remote-sensing data, we construct DNN models based on two classic, widely applied network frameworks—that is, U-Net [15] and SSD [17], respectively. The two frameworks are briefly introduced as follows.

### Framework of U-Net

**Figure 2.**
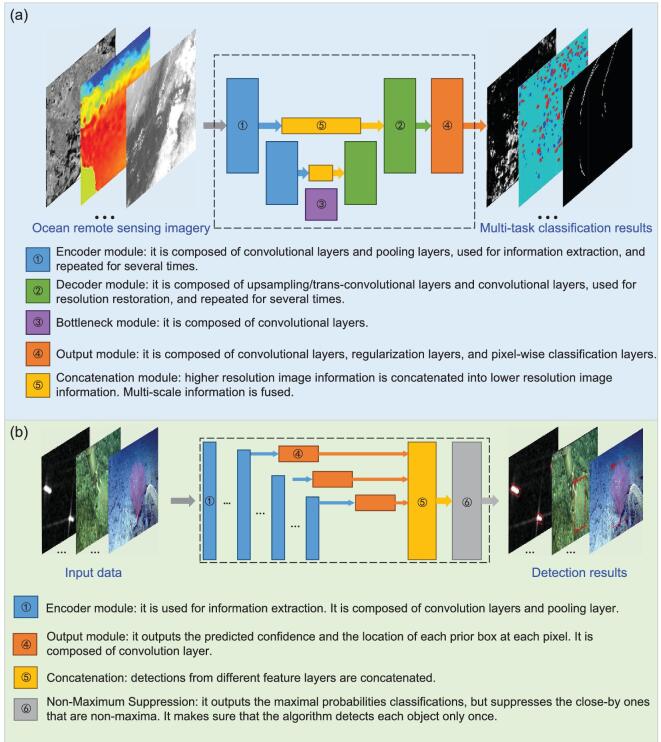
Moduled frameworks for pixel-level classification and object-level detection in ocean remote-sensing data. (a) The framework of U-Net. (b) The framework of single-shot detection (SSD).

Although developed initially for the semantic segmentation of biomedical images [15], U-Net achieves successful applications in many fields. U-Net is so named because of its almost symmetric encoder–decoder network architecture that can be depicted by a U-shaped diagram (Fig. 2a). U-Net uses the skip connection to pass the intermediate feature maps extracted by the encoder to the decoder. This idea helps to reduce the information loss caused by the resolution decrease in the data stream of the encoder.

U-Net extracts the features from an input image by outputting a class confidence for each pixel. The maximum confidence of a pixel indicates the class that the pixel is in. Then, the pixel-level classification map of the input data is generated.

As illustrated in Fig. 2a, U-Net consists of an encoder (left half in blue) and a decoder (right half in green). The encoder is used to extract image features at different resolutions. Along the data stream in the encoder, composite layers of cascaded convolutional layers alternate with max-pooling layers, and the feature-map resolution in the stream decreases after each max pooling. These composite layers can also be changed for more sophisticated ones (e.g. ResNet blocks [28]). The lowest-resolution feature maps extracted by the encoder are input into the decoder through the bottleneck at the bottom. Although those feature maps in which the grids have the largest receptive fields make the best use of the context in the input data, the more exceptional image features essential for the localization accuracy of semantic segmentation are lost due to the down-sampling caused by the max-pooling layers. To reduce the resolution loss, U-Net also passes the intermediate higher-resolution feature maps to the decoder by the skip connection (denoted by yellow modules in Fig. 2a). Contrary to the encoder, the decoder has an expansive data stream upward for resolution restoration. Its network architecture is almost the mirror of the encoder architecture, where the max-pooling operations are changed with up-sampling ones. The up-sampling operations can be realized by transposed convolutional layers or more efficient interpolation layers. After up-sampling, the decoded feature maps are concatenated with the encoder feature maps at the same resolution passed by the skip connection. Then, the concatenated feature maps are further decoded by the upper composite layer. The above procedure is repeated until the feature maps in the data stream have the same resolution as the input data. Then, these feature maps are processed by soft-max classification to yield the class confidence of each pixel.

Outlines of the object areas are delineated in raw image samples to generate pixel samples for the training of U-Net. Each pixel of the images is given a class label according to the outlines. Then, the label of each pixel is encoded into a one-hot vector that contains the ground-truth class confidence of the pixel. Such a pair of a pixel and its class confidence is a pixel sample.

The classification loss of U-Net is a soft-max loss, measuring the deviation of the output class confidences from ground-truth ones. The soft-max loss here is the sum of soft-max cross-entropy loss at each pixel. In the classic U-Net, to cause the contributions of some critical pixels to be large in the classification loss, a pixel-wise weight map is introduced in the soft-max loss, and the above sum is replaced with the weighted sum. The above essential pixels can be the pixels that are challenging to classify or the pixels of the classes having high importance but low occurrence frequency. The weight map can be used to balance the class-occurrence frequencies. An instance of a weight map is given in [16].

### Framework of SSD

SSD is a typical one-stage DNN for object detection without any fully connected layers [17]. SSD was proposed for efficiency. Tested on the VOC2007 data set, SSD run several times faster than the two-stage benchmark network Faster-RCNN [18] with a little higher accuracy in terms of mean Average Precision [17]. Different from other networks, SSD directly makes object detection in the multi-resolution feature maps of an input image, which enables SSD to detect objects at different scales.

SSD recognizes objects in an input image and outputs the rectangular areas (boxes) occupied by the objects as well as the confidences of the objects for different classes. The maximum confidence of an output box indicates the class that the object occupying the box is in. The output boxes are represented by their encoded center locations, widths and heights. The output boxes are redundant. Therefore, after the above representation is decoded, the output boxes are screened by non-maximum suppression to get the final boxes.

As shown in Fig. 2b, the backbone of SSD consists of two parts. The first part is to extract general features of objects from an input image, where feature-extraction modules of other networks can be adopted. In the classic SSD, VGG-16 [29] is used as the part and the fully connected layers in VGG-16 are replaced with convolutional layers. The second part is made up of several convolutional layers cascading the first part. The second part generates the multi-resolution feature maps. These multi-resolution feature maps progressively decrease in size with increasing network depth by a two-stride-step convolution. Accordingly, the receptive fields of the feature-map grids enlarge. SSD adopts a design similar to anchor boxes in Faster-RCNN [30]. Several boxes with different widths and heights are set at each grid of the multi-resolution feature maps. These boxes are called default boxes or prior boxes in SSD. The default boxes are also multi-resolution. That means that the default boxes of the feature maps at different resolutions have different scales relative to the input image. As the grid receptive fields enlarge, the box scales increase. The second part of the backbone has several network branches. The feature maps at different resolutions are further processed in the corresponding branches with small-kernel convolution, soft-max classification and bounding-box regression to get the class confidences, encoded center locations, widths and heights of the output boxes of each grid.

Ground-truth boxes and classes of objects are labeled in raw image samples to generate training samples of SSD. Then, intersection-over-union (IoU) is calculated between each ground-truth box and each default box. A boxed pair that has the maximum IoU of the ground-truth box in the pair is considered as a positive sample. The box pairs with IoU larger than a threshold (e.g. 0.5) are also considered as positive samples. Still according to the rule: a ground-truth box can match multiple default boxes in the positive samples, but a default box can only match one ground-truth box. The locations, widths and heights of the ground-truth boxes in the positive samples are encoded for the calculation of SSD loss. The rest of the default boxes are negative samples. Additionally, the strategy of hard-negative mining is also used to further balance the numbers of negative and positive samples. The strategy is that only the negative samples with the highest confidence loss are involved in the calculation of SSD loss rather than all the negative samples. The ratio between the negative and positive samples is at most 3:1.

The total SSD loss is a weighted sum of two components that are for bounding-box regression and classification, respectively. The first component is a smooth L1 loss to measure the loss between the encoded locations, widths and heights of the output boxes and those of the ground-truth boxes in positive samples. Another component is a soft-max loss to measure the classification loss, which is the sum of the soft-max cross-entropy losses of the samples used for training. The weight in the total SSD loss is usually set at one. Some detected objects (e.g. ships) have orientations. SSD can also be designed to have orientation-detection capability by adding the orientation angles of objects in the outputs and correspondingly modifying the first SSD loss component [31,32].

## APPLICATION EXAMPLES

In this section, we review the DNN-based supervised classification and object-detection applications in extracting several typical oceanic phenomena in ocean remote-sensing imagery. The applications include using geostationary satellite images for ocean internal wave information extraction; using SAR images for coastal-inundation mapping, sea-ice mapping, oil-spill mapping and ship detection; using the standard ocean remote-sensing AVISO (Archiving, Validation, and Interpretation of Satellite Oceanographic) SSH product for global ocean eddy detection; and using the MODIS (Moderate Resolution Imaging Spectroradiometer) images for Enteromorpha extraction. Using underwater-camera images, we also showed that the DNN-based model could be readily applied to extract coral-reef information from the seafloor.

### Internal-wave-signature extraction

The oceanic internal wave (IW) is a ubiquitous feature of oceans. It has attracted considerable research interest because of its essential role in ocean acoustics, ocean mixing, offshore engineering and submarine navigation [33–35]. Scientists have long recognized the potential for using satellite imagery for studying IWs [36–38]. Satellite images can compensate for the *in situ* observations to study the generation, propagation, evolution and dissipation of the IWs. In the past few decades, algorithms and techniques for automated detection of IW signatures from SAR imagery using basic image-processing methods have been studied significantly [39–41]. SAR is an active sensor that measures the sea-surface roughness. It is not affected by cloud cover and can image the ocean surface at a meter to tens of meters of spatial resolution under all weather conditions, day and night. The nature of SAR coverage is low. Therefore, scientists have also tried to extract IW information from geostationary satellite images that have lower spatial (250–500 m) but higher temporal (10 minutes) resolution under suitable solar-illumination conditions. Cloud cover and solar flares make the IW signatures much weaker and more challenging to extract from geostationary satellite images than that from the SAR images [42].

In recent years, machine-learning methods have been used widely to extract robust high-level information from satellite images. In this case, we applied a modified U-Net (Fig. 2a) framework to obtain IW-signature information in Himawari-8 images under complex imaging conditions.

**Figure 3.**
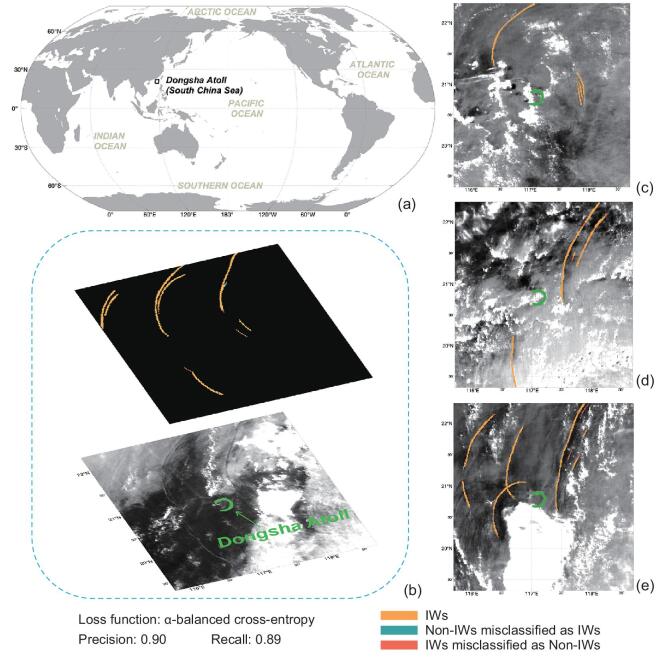
Four examples of the 40 testing results. (a) shows the study area. (b–e) are the input Himawari-8 images overlaid with their corresponding trained model extraction results. The four images were acquired at 05:40 on 30 May, 05:20 on 26 May, 06:00 on 21 May and 05:10 on 26 June 2018 (UTC), respectively.

The Himawari-8 satellite provides visible images of Earth's surface at a spatial resolution of 0.5–1 km and a 10-minute temporal resolution [43]. It is a useful tool to monitor and investigate the IWs in the South China Sea (SCS) [44]. We collected 160 Himawari-8 red-band images (1-km resolution) containing IW signatures around Dongsha Atoll in the northern SCS (Fig. 3) in May and June 2018.

The details of this modified U-Net framework is shown in Fig. 3. IW-signature extraction is essentially a binary pixel-wise classification problem. Typically, the loss function is a cross-entropy loss. However, in this IW-extraction case, IW only exists in very few pixels of the image, making the samples highly unbalanced and the use of cross-entropy loss invalid. To solve the class-imbalance problem, we adopted the *α*-balanced cross-entropy of Lin *et al.* [45] as the loss function and achieved excellent results (we set *α* to 0.99). To reduce the computation cost without losing generalization ability, we converted the images to the gray level and divided them into 256 × 256 pixel sub-images.

One hundred and twenty images, with their corresponding manually annotated ground-truth map for IW signatures (white) and surroundings (black), were randomly selected to train the U-Net framework, with the remaining 40 used as the testing images. After 200 epochs, the mean precision and recall of the testing set are 0.90 and 0.89, respectively.

Figure 3 shows the four examples from the 40 testing results. One can see that the sea-surface signatures of IWs were being not only overlaid with different types of clouds, but also strongly contaminated by solar flares as well as inhomogeneous darkness induced by other oceanic processes, all making the target signatures relatively weaker and difficult to extract. However, compared to the manually annotated ground-truth maps, the results from the U-Net model are good. Figure 3c captures a group of rarely observed reflected IWs propagating towards the east [46].

The statistical results (Fig. 3), relatively high mean precision and recall value of the 40 testing images showed that the U-Net framework is promising for the extraction of IW information in satellite images, even under complex imaging conditions.

### Coastal-inundation mapping

Tropical cyclone (TC)-induced coastal inundation is a typical compound natural hazard. It is the combination of storm-surge-caused inundation and heavy-rain-induced river flooding. TC-induced coastal inundation causes a huge loss of life and property in coastal areas [47–49]. Accurate mapping of coastal inundation from remote-sensing data can not only assist in the management of performing better disaster relief, but also help researchers to better understand the inundation mechanisms and develop more accurate forecasting models. SAR is a suitable sensing means for coastal inundation because it can provide day-and-night, all-weather observation abilities and high-resolution images of the flooded areas. The traditional ways of coastal-inundation mapping from SAR images include: histogram thresholding [50], active contour segmentation [51], region growing [52], change detection [53] and statistical classification [54]. The traditional methods are based on human-crafted features or rules to mine multidimensional SAR-image data for inundation mapping. It is difficult for them to provide robust performance under several influential factors: (i) the inherent speckle noise of SAR images; (ii) SAR-system factors; (iii) meteorological influences; and (iv) environmental influential factors.

Deep convolutional neural network (DCNN) models, composed of DNN-based models with convolutional layers, can provide a promising way to solve coastal-inundation-mapping problems. In the DCNN methods, the features for robust pattern classification for coastal-inundation mapping are mined from the multidimensional SAR data, instead of being predefined. This end-to-end, data-driven pattern-classification design is suitable for robust coastal-inundation mapping. Kang *et al.* [55] used the fully convolutional network model to verify that DCNN-based flooding detection is more accurate than the traditional methods. Rudner *et al.* [56] presented a DCNN-based method that is useful for flooded-building detection in urban areas. Liu *et al.* [57] proposed an improved DCNN method that has robust performance for coastal-inundation mapping from bi-temporal and dual-polarization SAR images.

To highlight the advantage of AI applications in coastal-inundation mapping, we constructed a DCNN framework to study this phenomenon. This framework can be generalized into studies involving multi-temporal SAR-information mining.

The framework is based on U-Net, as shown in Fig. 2a. The left part of the framework is the encoding part. It extracts abstracted features for accurate classification. It is composed of four encoding–decoding modules, as indicated by module 1/2 in Fig. 2a. Each encoding module includes two convolutional layers and one max-pooling layer. The right decoding part restores the feature-map resolution for pixel-level classification. Each decoding module includes one up-sampling layer and two convolutional layers. The input multidimensional SAR data are composed of pre-event, post-event and difference images of VH and VV polarizations (VH, vertical transmit and horizontal receive; VV, vertical transmit and vertical receive). This physics-based input information design fuses temporal and polarimetric information for more accurate coastal-inundation mapping. In the output module, as indicated by module 4 in Fig. 2a, a SpatialDropout2D layer is added before the classification to regularize the model for better generalization abilities [58]. Since inundation mapping is a binary classification problem, the binary cross-entropy is used for the loss function [45]. The detailed model design can be found in [57].

We used the 10-m-resolution Sentinel-1 SAR data to perform the experiments. We applied radiometric calibration, 7 *×* 7 boxcar filtering and geocoding to the SAR images. The ground-truth labels were extracted from Copernicus Emergency Management Service Rapid Mapping products [59] and human delineation with the help of Google Earth and OpenStreetMap.

**Figure 4.**
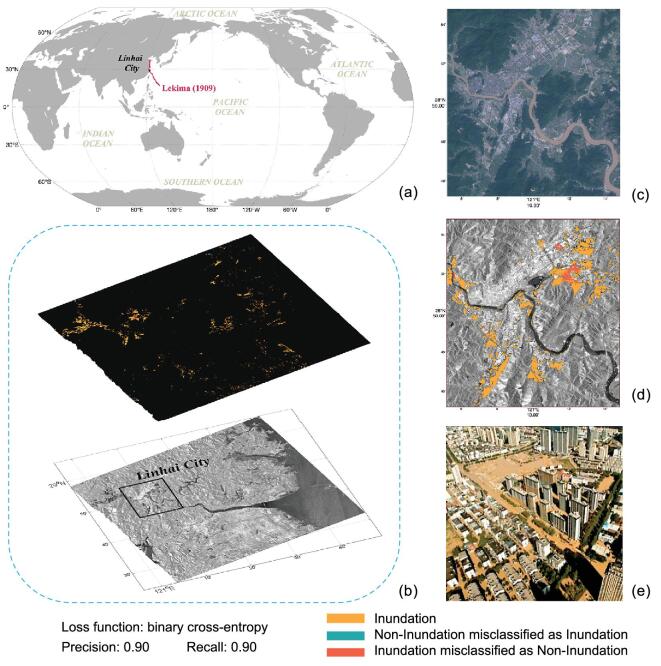
Deep-learning-based mapping result of Lekima-caused coastal inundation in Zhejiang, China. (a) The path of Lekima 2019. (b) The mapping result (upper image) and the pre-event SAR image (lower image) of the scene around Linhai city, Zhejiang. (c) The Sentinel-2 optical image of Linhai city. (d) The mapping result superimposed onto the SAR image in Linhai. (e) Picture taken after the passage of Lekima shows severe flooding in Linhai.

The training/testing samples were generated from 2017 Harvey-induced coastal inundation in Houston, Texas, USA and from 2019 Lekima-induced coastal inundation in Zhejiang, China, respectively. The 2019 Lekima path and the mapping result are shown in Fig. 4a and b. The orange areas are correctly extracted inundation. The red areas are missed detections and the cyan areas are false alarms. The precision and recall of the result are both around 0.90. The result shows the severe inundation in Linhai city, Zhejiang, as illustrated in Fig. 4d. The Sentinel-2 optical image of Linhai is shown in Fig. 4c. The picture taken after the passage of Lekima confirms that Linhai was severely flooded, as shown in Fig. 4e.

The AI technology, particularly the DCNN methods, can mine multidimensional SAR data for accurate and robust coastal-inundation mapping. In the future, the model can be extended to work under multiple image-source conditions for more practical applications.

### Global mesoscale-eddy detection

Mesoscale eddies are circular currents of water bodies that widely exist in the global oceans. They play a significant role in the transport of momentum, mass, heat, nutrients, salt and other seawater chemical elements across the ocean basins. They also impact global ocean circulation, large-scale water distribution and biological activities effectively [60–63].

Automatic eddy-identification algorithms include the physical-parameter-based method [64–66], the flow-direction-based method [67] and the SSH-based method [61]. All these algorithms lack the computational efficiency of contour iterations or have complex calculation processes.

In the introduction part, we described that the DNN-based framework can solve many practical problems such as pattern recognition and computer vision with high efficiency. It is natural to propose using the DNN framework to detect mesoscale eddies that have prominent patterns in the global SSH maps. In the literature, Lguensat *et al.* [68] developed ‘EddyNet’ that was based on the encoder–decoder network U-Net (Fig. 2a) to detect oceanic eddies in the southwest Atlantic. Franz *et al.* [69] also use the U-Net architecture to identify and track oceanic eddies in Australia and the East Australia current. Du *et al.* [70] developed ‘DeepEddy’ based on PCAnet (Principal Component Analysis Network) and spatial pyramid pooling to detect oceanic eddies based on SAR images. Xu *et al.* [71] applied the pyramid scene parsing network ‘PSPNet’ to catch eddies in the North Pacific Subtropical Countercurrent region. These regional studies proved that the DNN performed well in detecting mesoscale eddies in territorial seas. The DNN performance on global mesoscale-eddy detection remained unverified.

In this section, we applied a generalized DNN framework to detect global mesoscale eddies. The framework is based on the U-Net architecture (Fig. 2a) consisting of ResNet blocks. Each ResNet block is composed of a 3 × 3 convolutional layer, followed by a batch normalization (BN) and a rectified linear unit (ReLU) activation, then a 3 × 3 convolutional layer and a BN layer. The output is added to the input to be activated by a ReLU layer. For the encoder path, each block is followed by 2 *×* 2 max pooling and dropouts. For the decoder path, transposed convolutions are used to restore the original resolution. The dice loss function, which is widely used in segmentation problems, is the cost function. The 0.25° *×* 0.25° spatial resolution and daily SSH product during 2000–11 was generated by Ssalto/Duacs and distributed by AVISO. Mesoscale eddies that identified by the SSH-based method [61] during the same period were used as the ground-truth data set. Pixels were labeled as ‘1’, ‘−1’ and ‘0’ inside anticyclonic eddies (AEs), cyclonic eddies (CEs) and background regions.

**Figure 5.**
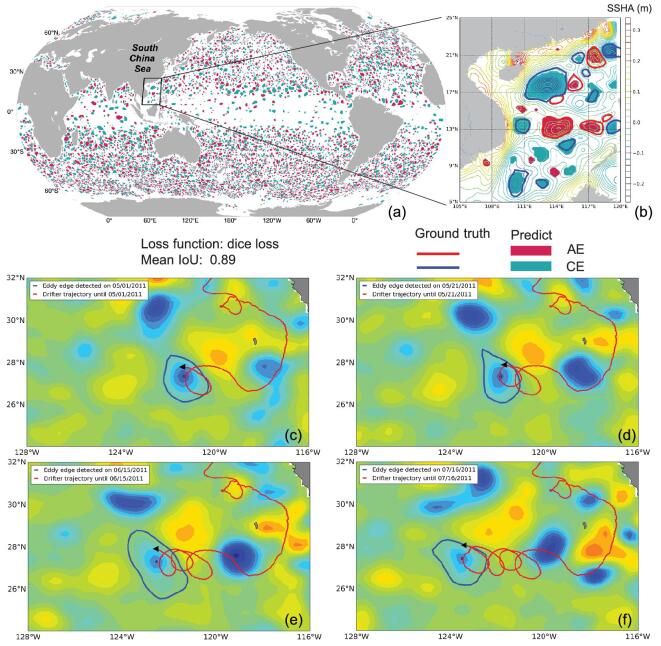
(a) Mesoscale eddies detected by the AI-based method in the global ocean on 1 January 2019. (b) Mesoscale eddies detected by the SSH-based method and AI-based method in the SCS on 1 January 2019. A drifter is captured by a CE that was detected by the AI-based method in the eastern North Pacific and rotated with the CE on (c) 1 May 2011, (d) 21 May 2011, (e) 15 June 2011 and (f) 17 July 2011 (the color denotes SSHA — sea surface height anomaly).

Figure 5a shows the mesoscale eddies identified by the DNN method on 1 January 2019. There are a total of 3314 (2963 ground-truth) AEs and 3407 (3056 ground-truth) CEs in the global ocean. Compared to the SSH-based method, the accuracy of the DNN-based eddy-detection method is 93.79% and the mean IoU is 88.86%. Figure 5b clearly shows that the DNN-based framework identified many more small-scale eddies. Besides, it takes <1 minute for the DNN-based method costs to identify eddies in the global ocean, while the SSH-based method takes >16 hours [72].

The satellite-tracked drifter is used to validate the eddy-detection results of the DNN-based method. The drifters have their drogues centered at 15-m depth to measure the surface currents and make either a cycloidal or a looping trajectory when trapped in an eddy. As shown in Fig. 5c, a drifter (ID: 43677) was trapped in a CE in the eastern North Pacific on 1 May 2011. After 20 days, the drifter captured in the CE moved as a counterclockwise loop (Fig. 5d). Another two counterclockwise loops of drifter trajectory can be seen in the CE in Fig. 5e and f, after 25 and 30 days, respectively. Such a result is consistent with the concept that CEs rotate counterclockwise in the Northern Hemisphere.

In conclusion, the DNN-based eddy-detection method can not only identify many more small-scale eddies, but also significantly improve the computational efficiency. Further development of the DNN-based framework includes adding other types of ocean remote-sensing images, i.e. SST, chlorophyll concentration, etc., to a multi-parameter-based DNN framework.

### Oil-spill detection

Oil spill is typical marine pollution. Oil floating on the sea and beaching on the shore could seriously affect surrounding marine fisheries, aquaculture, wildlife, ecosystems, maritime tourism and transportation, among others. For example, the Deepwater Horizon (DWH) oil spill is an example of severe marine-pollution disasters. It happened in the Gulf of Mexico on 20 April 2010 and an estimated 7.0 × 10^5^ m^3^ of oil was released into the sea before the well was capped on 15 July 2010 [73]. Accurate detection of oil spills from remote-sensing data can help disaster-relief managers to perform targeted responses and it can also assist scientists in forecasting the movement and dissipation processes of oil spills. SAR is a suitable sensor for oil-spill detection because the oil dampens the sea-surface capillary waves so that they appear dark in SAR intensity images [74–76]. Besides, oil slicks also modulate the surface-scattering matrix received by advanced polarization SAR [77–83]. As a result, oil slicks also have significant signatures in the full-polarization SAR images.

In the big-ocean-data era, AI technology has the potential to mine information from a high volume of polarization SAR images acquired under different meteorological conditions and system parameters. It is promising to develop a robust feature-detection algorithm using such technology. For example, Chen *et al.* [84] used the DNN framework to optimize the polarimetric SAR feature sets to reduce the feature dimension for oil-spill detection and classification. Guo *et al.* [85] proposed a DNN-based polarization SAR-image-discrimination method for oil slicks and lookalikes. Guo *et al.* [86] used the DNN-based semantic-segmentation model to detect oil spills by using backscattering energy information.

To demonstrate that the AI technology has great potential for robust oil-spill detection and characterization under various meteorological and SAR-acquisition conditions, we constructed a generalized AI framework to study oil detection in polarization SAR data.

The framework is based on U-Net, as shown in Fig. 2a. The left encoding part extracts abstracted features with four encoding modules, each including two convolutional layers and one max-pooling layer. The right decoding part restores the feature-map resolution with four decoding modules, each including one up-sampling layer and two convolutional layers. The input configuration is to use the diagonal components of the polarimetric coherence matrix [87], T11, T22, T33, with and without the incidence angle. In the output module, a Spatial-Dropout2D layer [57] is added before the classification to regularize the model for better generalization. Since oil-spill detection is a binary classification problem, we selected the binary cross-entropy as a loss function [45].

We applied this DNN-based model to a set of L-band Uninhabited Aerial Vehicle SAR (UAVSAR) images taken during the DWH oil-spill event by the National Aeronautics and Space Administration (NASA) [88]. The UAVSAR is a full-polarization SAR with fine resolution (7 m), stable calibration and low noise floor. Radiometric calibration and 7 × 7 boxcar filtering were applied to the data. To show the incidence angle's influence, geocoding was not used. According to the detailed analysis with *in situ* observations of [89], we manually extracted ground-truth labels. The training/testing samples were from different UAVSAR images with flight ID 14010/32010.

**Figure 6.**
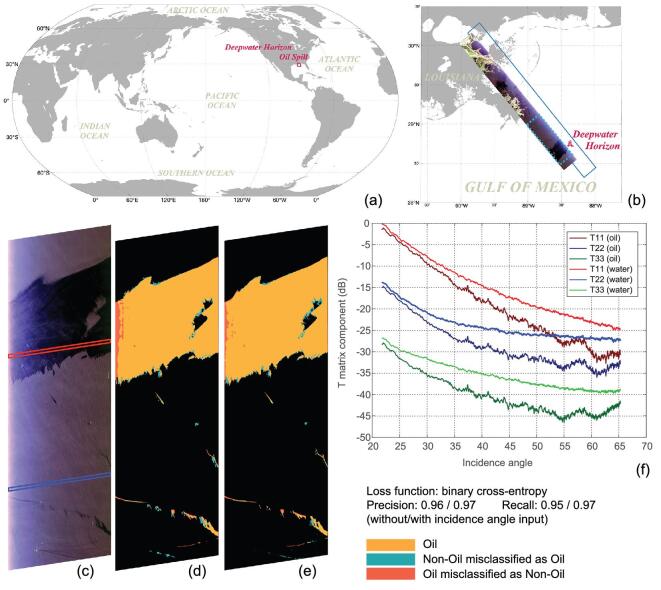
Deep-learning-based mapping result of the Deepwater Horizon (DWH) oil spill in the Gulf of Mexico. (a) The DWH oil spill in the world map. (b) The polarimetric SAR images used in the training and testing. The polarimetric SAR image in the dashed blue rectangle was used for testing and performance evaluation. (c) The PauliRGB pseudo-color image of the testing area. (d) The deep-learning-based result without the incidence angle. (e) The deep-learning-based result with the incidence angle. (f) The relationship of the polarimetric coherence matrix components and the incidence angle of the oil and water areas in (e).

Figure 6 shows the testing image with PauliRGB pseudo-color [87]. From left to right, the incidence angle increases from 22° to 65°. We can observe that the incidence angle has an impact on the oil–water discrimination capability. An oil area (in the red rectangle) and a water area (in the blue box) were selected to show the trends of the T11, T22 and T33 components with incidence angle, as illustrated in Fig. 6c. We can see that the incidence angle influences the oil–water discrimination capability. Below 30°, it is challenging to discriminate oil from water. Moreover, according to [89], the backscattered information is influenced by the noise floor, especially above 60°. The detection results without and with incidence angle are shown in Fig. 6d and e, respectively. The correctly detected areas are in orange, the missed detections are in red and the false alarms are in cyan. The recall and precision of the result without using the incidence angle are 0.95 and 0.96, respectively. The recall and accuracy of the result with the incidence angle are both improved to 0.97. With the incidence angle in training and testing, the AI technology can mine reliable features for robust pattern classification, even with a minimal incidence angle.

AI technology, particularly the DCNN methods, can mine multi-polarization SAR data for accurate oil-spill detection. It shows the potential for robust detection under various influential factors. In the future, a more extensive range of factors should be considered and analysed for practical use.

### Sea-ice detection

Sea ice is a significant threat to marine navigation and transportation safety. The change of sea-ice distribution reflects the interaction of the atmosphere–cryosphere–hydrosphere and global climate change. Sea-ice detection and monitoring draw attention widely. SAR, which is independent of sun illumination and cloud conditions, plays an vital role in sea-ice monitoring [90]. A series of studies have been devoted to the SAR sea-ice-detection problem. The critical challenge is to develop a robust model that captures domain-specific expert knowledge for discriminating between ice and water using SAR backscatter characteristics. To achieve this goal, different types of sea-ice-detection models based on backscatter thresholding [91], regression techniques [92], expert systems [93], Bayesian techniques [94], gray-level co-occurrence matrix and the support vector machine (SVM) hybrid method [95], among others [96], are proposed.

Recently, with the rapid progress of AI technology, researchers have employed DNN to extract features automatically to improve the accuracy and efficiency of sea-ice classification. Xu and Scott [97] introduced an earlier CNN-based model AlexNet and transferred learning to classify sea ice and open water. Gao *et al.* [98] integrated transfer learning and dense CNN blocks to form a transferred multilevel fusion network (MLFN). The MLFN outperformed the PCAKM [99], the NBRELM [100] and the GaborPCANet [101] in classifying sea ice and open water. Similar DL-based studies were carried out by other researchers [102,103]. More and more researchers are trying to construct DL-based models to achieve end-to-end sea-ice detection with higher accuracy and stability.

To highlight the advantage of AI applications in sea-ice classification, we constructed a generalized AI framework based on U-Net (Fig. 2a). The input was a 256 × 256 SAR image. We stacked four encoder modules to extract features level by level. Each encoder was composed of two convolutional layers with ReLU units followed by a max-pooling layer. One bottleneck module that was composed of two stacking convolutional layers with ReLU units was added onto the last encoder module. Four decoder modules were stacked upon the bottleneck module. Each decoder module was composed of one up-sampling layer and two stacking convolutional layers with ReLU units. The concatenation module was applied to fuse the encoder module and the decoder module at the same level. The output module consisted of one CNN layer with one activation layer, which outputted the predicted value of each pixel. We transformed the detection procedure as a binary classification problem: sea ice or open water. Thus, we applied sigmoid as the activation function. If the predicted value is >0.5, the pixel belongs to sea ice. Otherwise, the pixel is open water. The loss function is binary cross-entropy.

To show the effectiveness of this AI model, we acquired six Sentinel-1 SAR images in the Bering Strait between February and April 2019. The 10-m-resolution VV channel of the ground range detected SAR product was selected as the experiment data set. We scaled all SAR pixel values between 0 (water) and 1 (sea ice). Each SAR image was divided into small chips with a size of 256 × 256 pixels.

**Figure 7.**
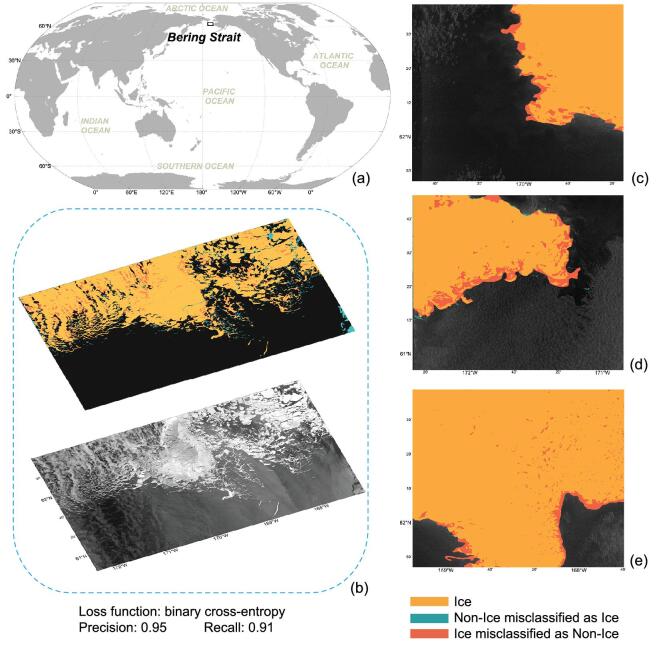
Testing results of the proposed sea-ice-detection framework. (a) The overall location of the study area. (b) Detection results of the first testing SAR image. (c–e) Detection results of the second testing SAR image.

There were 1340 chips in the training set. The batch size was 16. The initial learning rate was 0.0001. We split 20% samples from the training set as the validation set. The early stopping strategy was adopted to avoid overfitting. Finally, the model ran 86 training epochs. The precision and the recall of the testing set were 0.95 and 0.91, respectively. As shown in Fig. 7b–e, most of the sea ice, including small chunks and sinuous ice edges, could be successfully detected. However, the rough sea surfaces resulted in some misclassifications, which need to be further addressed.

The proposed U-Net-based model is capable of detecting sea ice from SAR images at the pixel level. The detection framework is an end-to-end model without manual feature engineering and expert knowledge. The detection results (Fig. 7) show that details such as the boundary between sea ice and open water can be successfully detected. In the future, employing DNN-based models to detect or estimate more parameters of sea ice, such as the type, the thickness and the intensity, etc., should be new challenges.

### Green-algae detection

**Figure 8.**
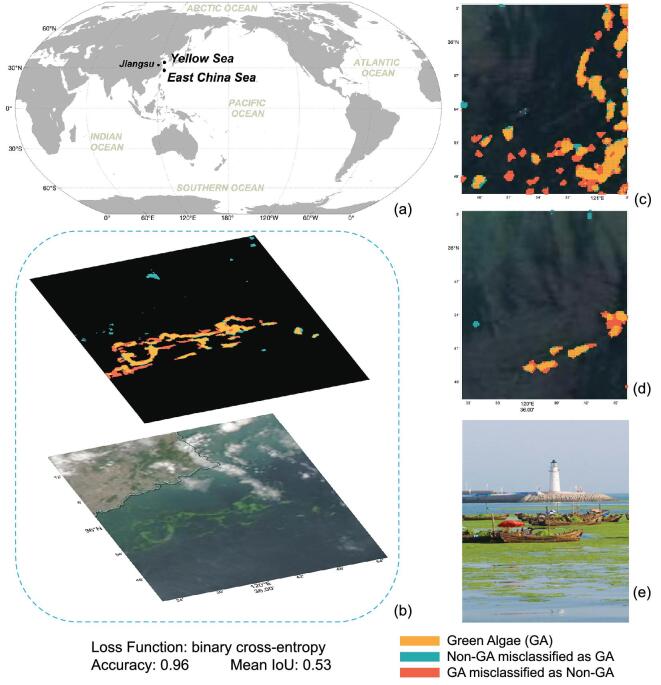
*Enteromorpha prolifera* (a type of green algae) detection. (a) Study area. (b) MODIS images and its corresponding *Enteromorpha prolifera* classification result. (c) and (d) The fallout ratio or the omission ratio in some pixels due to the cloud contamination of the optical images. (e) Picture taken after *Enteromorpha prolifera* bloom.

*Enteromorpha prolifera* (EP), as a kind of large-sized green algae, is fulminant and drift in the East and Yellow Seas of China in spring and summer seasons. Sporadic EP first occurred along the coast of Jiangsu province at about 35°54*^′^* in Fig. 8a, then drifted northward driven by the wind and current. During the drifting process, when meeting the appropriate water-quality conditions, large-scale proliferation and aggregation occurred. Since 2008, a high concentration of EP has beached along the coast every year, causing a so-called green-tide disaster affecting ship traffic, the environment, coastal ecosystems, public health and the tourism industry, among others [104,105]. Mapping and tracking EP in near real time facilitates treatment processing.

EP has distinguished features in sea-surface-reflectance images acquired by the MODIS sensors on board NASA Aqua and Terra satellites. The aggregation of seaweed and its decomposition alter the water-surface-reflectance values [106]. For EP and other types of floating green-algae detection in ocean remote-sensing images, multiband ratio methods, e.g. NDVI (normalized difference vegetation index [107]) and FAI (floating algae index [108]) were developed. In general, these methods have reasonable visual interpretation and low error rates.

As we pointed out in the introduction part, DNN has significant superiority over the physical-based algorithms for image classification. For example, Arellano-Verdejo *et al.* [109] proposed a DNN framework model named ‘ERISNet’ to classify the presence/absence of pelagic sargassum along the coastline of Mexico. The model is based on a 1D CNN and achieves a probability of 90.08% in the classification at the pixel level. In this section, we customized an EPNet model based on the U-Net framework (Fig. 2a) for EP classification in MODIS imagery. The significant difference between ‘ERISNet’ and ‘EPNet’ is that we used 2D convolution in the DNN model. The intermediate architecture consists of symmetrical ascending and descending structures, which include five encoder and decoder modules, respectively, and the binary cross-entropy is the loss function.

We manually extracted EP labels from the MODIS true-color images (bands: 1/4/3). To construct a labeled data set, we collected different types of tags (banded, lumpy and dotted types) under different environmental conditions. We also followed the common practice of expanding the sample size by rotation of the sample images by 90°, 180° and 270°. For MODIS images acquired between 2008 and 2019, we obtained 1680 pairs of MODIS EP slices and their corresponding labels (128 × 128 pixels). A randomly selected 1460 and 220 pairs were used as training and testing sets, respectively.

Figure 8a shows the EP blooms in the Yellow Sea of China. EPNet achieves an overall classification accuracy of 0.96 with a mean IoU of 0.53 (Fig. 8b). However, there are some fallout ratios or omission ratios in some pixels in Fig. 8c and d. The misclassification is due to the cloud contamination of the optical images. Nevertheless, our analysis shows that the DNN framework can be readily implemented to identify EP.

### Ship detection

Ship detection plays a significant role in marine surveillance. SAR has been widely used in marine-ship detection because it is capable of monitoring ocean targets under all weather conditions, day and night [110–112]. For decades, a series of studies have been devoted to detecting ships and other targets in SAR images. The algorithms can be divided into conventional methods and AI-based methods. A typical conventional method is threshold-based methods that focus on finding bright pixels operating accurate clutter statistical modeling. Algorithms built on the theory of CFAR filtering [10] and generalized likelihood ratio testing (GLRT) [113] are representations. The main drawback of the conventional methods is that they need prior professional knowledge to manually design features, which has been a common challenge faced by most fields in the era of big data [11].

The cutting-edge AI framework, deep learning, can extract features automatically, which has been a great achievement in computer vision. Faster region-based convolutional network (Faster-RCNN) [18] is a complete end-to-end CNN target-detection model. Researchers have introduced Faster-RCNN to detect ships in SAR images [114]. Other studies tried to introduce rotatable bounding boxes into detection models to represent ships more accurately. Liu *et al.* [115] proposed a detector using rotatable bounding boxes (DRBox), which optimized the traditional SSD [17] by rotating the prior box. DRBox outperformed traditional SSD and Faster-RCNN in detecting densely arranged ships. Other rotated detectors such as rotation dense feature pyramid networks (R-DFPN) [31] and DRBox-v2 [116] were proposed successively. Notably, DRBox-v2 improved DRBox by integrating new DL tricks such as a feature pyramid network (FPN) [117] and focal loss (FL) [45], which outperformed R-DFPN and DRBox in detecting ships and airplanes [116]. Compared with conventional methods, the DNN-based ship-detection models significantly simplify feature engineering and achieve end-to-end detection with higher accuracy and stability.

To highlight the advantage of AI applications in ship detection, we constructed a generalized AI framework based on SSD (Fig. 2b) to fulfill the task.

The input was a 300 × 300 pixels SAR image. We stacked five encoder modules to extract features. The output module performed convolution on feature maps and generated the location and the confidence of the detected boxes. Different from DRBox-v2, the detected boxes were generated based on the last four feature maps (DRBox-v2 is the last three). The new added shallow feature map helps to detect small targets. FPN was adopted to fuse features at different levels and the rotation box was adopted. The loss function (*L*) consists of two parts: the confidence loss (*L_conf_*) and the location loss (*L_loc_*):

(1) \begin{equation*} L{\rm{\ }} = \frac{1}{{{N_1} + {N_2}}}{\rm{\ }}{L_{\textit{conf}}} + \frac{1}{{{N_2}}}{L_{\textit{loc}}}, \end{equation*}

where *N*_1_ is the number of negative samples and *N*_2_ is the number of positive samples. *L_conf_* is the cross-entropy between the output and the ground truth, which can be defined as follows:

(2) \begin{equation*}{L_{\textit{conf}}} = \ - \mathop \sum \limits_{i \in \textit{Pos}} \log\! \left( {{c_i}} \right) - \mathop \sum \limits_{i \in \textit{Neg}} \log ( {1 - {c\!_j}}), \end{equation*}

where *c_i_* is the confidence of the *i_th_* positive samples and *c_j_* is the confidence of the *j_th_* negative samples; *Pos* and *Neg* are positive and negative sets, respectively. Hard-negative mining (HNM) [17] and FL are employed to overcome the problem of the imbalance between the positive and negative samples. For location loss, *L_loc_* measures the location difference between the predicted rotation bounding box and the matched ground truth, which can be calculated as follows:

(3) \begin{eqnarray*} {L_{\textit{loc}}} &=& \mathop \sum \limits_{i \in {\textit Pos}} \mathop \sum \limits_{j \in {\textit {Grd}}} \mathop \sum \limits_{k \in \left\{ {x,y,l,w,\theta } \right\}} {I_{\mathit{ij}}}\,{\textit{smooth}_{L1}}\nonumber\\&&\times\left( {p_i^k - g_j^k} \right), \end{eqnarray*}

where *k* is the location vector consisting of center coordinates (*x, y*), length (*l*), width (*w*) and angle (*θ*) of the box. *p_i_* is the prediction location of the *i_th_* positive box and *g_j_* corresponds to the *j_th_* ground truth. *smooth_L_*_1_ means *L*1 norm. *Grd* is the ground-truth sets. *I_ij_* means if the *i_th_* prior box matches the *j_th_* ground truth: *I_ij_* = 1 when matching, otherwise *I_ij_* = 0.

The OpenSARShip [118], a data set dedicated to Sentinel-1 ship interpretation, was employed as the sample data set. We labeled the ship by a rotation bounding box with a MATLAB tool shared in DRBox-v2.

**Figure 9.**
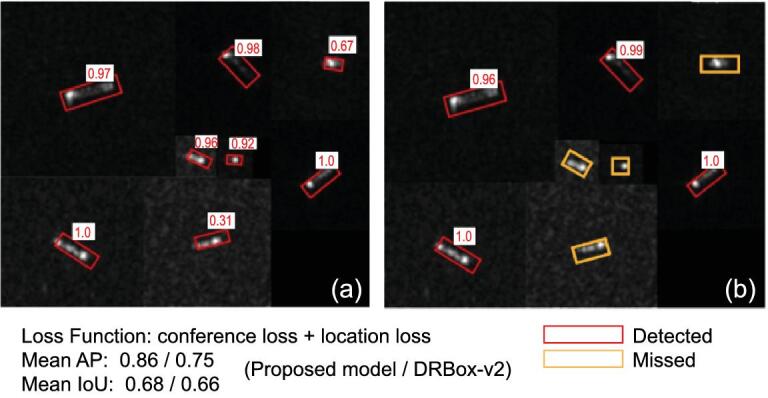
Testing results of the proposed ship-detection framework. (a) Detection results of the proposed optimized DRbox-v2 model. (b) Detection results of the original DRbox-v2 model. The red rectangle represents correct detection and the orange rectangle represents missed detection. The number in each white box represents the detection confidence.

The training and testing sets included 1600 and 338 ship chips, respectively. The batch size was eight. After 8200 training epochs, the loss value was <0.01 and the model stopped. Finally, 297 ships were successfully detected by the proposed model. The mAP (mean Average Precision) and mean IoU were 0.86 and 0.68, respectively. The original DRBox-v2 detected 267 ships and its mAP and mean IoU were 0.75 and 0.66, respectively. As shown in Fig. 9, some ships that were not successfully detected by the original DRBox-v2 model were successfully detected by the optimized one.

The proposed SSD-based framework is capable of detecting ships from Sentinel-1 images. By adding a shallow feature map, the detection accuracy was improved significantly. In the future, constructing end-to-end AI-based models to identify the type and geometric parameters of ships from SAR images should draw more attention.

### Coral-reef detection

Marine species are a vital part of the ocean and play an essential role in the trophic chain of the ecosystems. Detection and identification of marine species are one of the crucial ways to explore marine biodiversity. Traditional methods of biometric identification are based on morphology and molecular genetics, or even the start-of-the-art DNA (deoxyribonucleic acid) sequencing under electron microscopy in the laboratory. Although these methods are accurate, we can not carry out such experiments in the actual marine environment. Another issue is that *exsitu* detection often causes organisms to become inactive or die.

To solve these issues, we can apply AI-based methods to detect marine species on the fly. As we have shown in previous sections, DNN has a significant advantage in satellite-image classification. The same technology can also be applied to underwater-camera-image classification. Recently, Villon *et al.* [119] used the CNN framework to detect and classify fish and they showed the CNN outperformed the traditional SVM classifier. Xu *et al.* [120] presented a comprehensive review of the computer-vision techniques for marine-species recognition from the perspectives of both classification and detection. They further compared the machine-learning techniques with deep-learning techniques and discussed the complementary issues between these two approaches. Using a new genetic-programming approach, Marini *et al.* [121] achieved high reliability when tracking fish variations at different time scales but failed to classify fish while monitoring. Saqib *et al.* [122] adopted an end-to-end Faster-RCNN for surveillance and population estimation of marine animals. Faster-RCNN significantly improves the mAP, but the detection speed is prolonged. Pedersen *et al.* [123] used the YOLOv3 (You only look once) [124] framework and the Brackish Dataset to detect big fish, small fish, crab, jellyfish, shrimp and starfish.

To highlight the advantage of AI applications, we constructed a generalized AI framework based on SSD (Fig. 2b) for coral-reef detection in underwater-camera images. SSD is a one-stage classifier. We can train an SSD model by simultaneously optimizing classification loss and localization loss. Compared with the two-stage classifier, SSD is much faster while still ensuring classification accuracy [17]. As a result, SSD makes real-time underwater-species detection and classification possible. Our SSD framework is based on VGG16 [29], which is pre-trained on the ILSVRC CLS-LOC data set [125]. We converted fc6 (the sixth fully connected layer) and fc7 to convolutional layers, subsample parameters from fc6 and fc7 and after that removed all the dropout layers and the fc8 layer using SSD Weighted Loss Function [17]. We adjusted the outcome model using stochastic gradient descent (SGD) with an initial learning rate of 0.0004, 0.9 momenta, 0.0005 weight decay and a batch size of 32.

The experimental marine organisms included *Chrysogorgia ramificans* [126], *Chrysogorgia binata* [126], *Paragorgia rubra*, [127] and *Metallogorgia macrospina*, which were collected by RV (research vessel) *KEXUE*. We split videos of these four species into frames and annotated pictures manually. In the data preprocessing, we referred to the Australian benthic data set [128]. We randomly divided the samples into training, validation and test according to a certain proportion. To make the SSD-based model robust, we selected training-set images containing different types of species collected under different underwater conditions and shooting angles. Data preprocessing included two parts: data-format conversion and image-size standardization.

**Figure 10.**
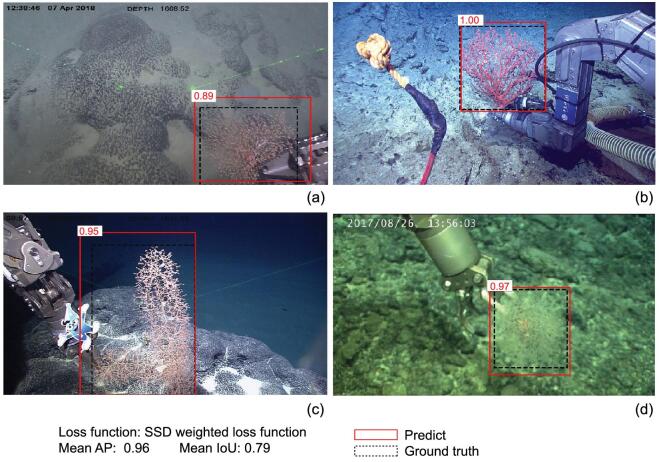
(a) *Chrysogorgia binata* [126], (b) *Paragorgia rubra* [127], (c) *Chrysogorgia ramificans* [126], (d) *Metallogorgia macrospina*. The number in each white box represents the detection confidence.

We applied the SSD-based model to 59 test images. Among all the four different coral species, Fig. 10 shows that the SSD-based model achieved 0.96 mAP with an average IoU value of 0.79. This result is remarkable and the SSD-based model can be used in coral-reef classification in real time. To further detect small-sized coral reef, we need to increase the sample size and species categories.

## CONCLUSION AND FUTURE PERSPECTIVES

Ocean remote sensing has entered the big-data era with typical five-V characteristics. In the age, ideal data-mining technology should be able to extract sparse but valuable information from enormous ocean remote-sensing data volumes precisely, efficiently and with very little human involvement. The technology should also be smart and robust enough to cope with various problems that ocean remote-sensing big data contain. The above requirements can be summarized into three Hs (high precision, high efficiency and high intelligence). Emergent deep-learning technology satisfies the three-H requirements and provides a promising way for such information extraction.

Pixel-level classification and object-level detection are two fundamental tasks in information extraction. As a result, we introduced two representative DL frameworks (U-Net and SSD) to demonstrate the powerful capability of DL technology to fulfill these tasks.

Although DL is a potent tool and demonstrates its advantages for information mining from ocean remote-sensing imagery, we still need to consider some key issues when we look ahead.

First, the DL-based technology is data-hungry. Notably, the DL-based technology needs enormous amounts of highly accurate labels. Currently, objects of interest are usually manually labeled and the label accuracy is subject to human experience and errors. As a result, the DL model trained with those labels inevitably introduces errors into the output results (e.g. statistics of shapes, sizes and areas of objects). For DL technology to reveal oceanic reality, we should provide labels from reliable *in situ* measurements, which requires world-level collaborations. Standard data sets with joint efforts of the entire community should boost the DL-based ocean remote-sensing imagery-information mining. If big data are the door to AI ocean remote sensing, then DL technology is the key to the door. For some studies, we still rely on expert knowledge to provide the ground truth. It is essential to combine the knowledge of different expert groups to eliminate human bias. One possible solution is to develop unsupervised DL methods that avoid the limitations of human knowledge.

Second, most DL models for ocean remote-sensing imagery-information mining come from the computer-vision community. These models are developed initially to extract spatial and temporal patterns to solve vision problems. These models should and could be guided and specially tailored to serve the purpose of ocean-science applications. To tackle a specific problem, the combination of the knowledge of big-data scientists and particular domain scientists would help to reveal the real world more effectively than ever before.

Third, for DL-based ocean remote-sensing imagery-information mining, the trained DL models are often sensitive to sensors, as shown in this study. If we train these different models for different sensors, it is computationally expensive and labor-costly. We need to study practical ways of transferring models from one sensor to another and improve the model's generalization capability under different sensing conditions.

In this paper, we reviewed eight typical DL framework applications in ocean internal-wave/eddy/oil-spill/coastal-inundation/sea-ice/green-algae/ship/coral-reef mapping from different types of ocean remote-sensing imagery. We described the general deep-learning model set up for data mining in ocean remote sensing and showed that the U-Net and SSD models achieved superior performances in these topics.
